# High Prevalence of Human Liver Infection by *Amphimerus* spp. Flukes, Ecuador

**DOI:** 10.3201/eid1712.110373

**Published:** 2011-12

**Authors:** Manuel Calvopiña, William Cevallos, Hideo Kumazawa, Joseph Eisenberg

**Affiliations:** Universidad Central del Ecuador Centro de Biomedicina, Quito, Ecuador (M. Calvopina, W. Cevallos);; Kochi University School of Medicine, Kochi, Japan (H. Kumazawa);; University of Michigan, Ann Arbor, Michigan, USA (J. Eisenberg)

**Keywords:** Amphimerus, Ecuador, South America, liver fluke, parasites, trematoda, human infection, zoonoses

## Abstract

*Amphimerus* spp. flukes are known to infect mammals, but human infections have not been confirmed. Microscopy of fecal samples from 397 persons from Ecuador revealed *Opisthorchiidae* eggs in 71 (24%) persons. Light microscopy of adult worms and scanning electron microscopy of eggs were compatible with descriptions of *Amphimerus* spp. This pathogen was only observed in communities that consumed undercooked fish.

The genus *Amphimerus* Barker 1911 infects mammals from the Americas, including Canada, the United States, Costa Rica, Panama, Colombia, Brazil, and Peru. Eleven species are reported (*1**–**7*). In Ecuador, a trematode resembling *Amphimerus* spp. but identified as *Opisthorchis guayaquilensis* has been reported (*8**,**9*).

*Amphimerus* spp. are parasitic liver flukes in the bile ducts of mammals, birds, and reptiles (*1*). Although these digenetic trematodes of the *Opisthorchiidae* family are closely related to the genera *Clonorchis* and *Opisthorchis*, there are morphologic differences*.* The vitellaria in adult *Amphimerus* spp. trematodes are distributed in 4 groups, 2 anterior and 2 posterior; the latter groups extend beyond the posterior testis; the ventral sucker is larger than the oral, and the testes are rounded or slightly lobulated. In contrast, the vitellaria in *Clonorchis* and *Opisthorchis* spp. worms exist only in front of the testes. Additionally, *Clonorchis* spp. trematodes have 2 large highly branched testes; testes in *Opisthorchis* spp. trematodes are always lobulated (*1**,**2*). The eggs of the flukes from these genera can be differentiated only by using scanning electron microscopy (SEM). Definitive diagnosis using light microscopy of the flukes of the *Opisthorchiidae* family, therefore, is not possible unless the adult worm is collected and identified. Through isolation of adult worms and SEM of eggs, we found a high prevalence of human infection with a trematode of the genus *Amphimerus* in Ecuador.

## The Study

In June 2009, during a routine fecal examination for the parent study, 4 samples tested positive for eggs of the *Opisthorchiidae* family in 3 indigenous Chachi communities along the Cayapas River in the northern coastal rainforest of Ecuador. In January 2010, a follow-up survey was conducted in the same 3 communities (total population 589); all villagers, whether symptomatic or not, were asked to provide a fecal sample. Specifically, a community meeting was held in each village, study objectives were explained, and villagers were asked for their voluntary participation. Flasks were distributed to all villagers and collected the next day in the school and by going house to house. The Chachis, the predominant group in these 3 communities, represent 13% of the 24,000 inhabitants in the region. Afro-Ecuadorians and mestizos also reside in this region (*10**,**11*).

A total of 297 (50.4%) community members 3–77 years of age provided samples. To each person providing a sample, a questionnaire was administered regarding types of food eaten and cooking practices. Samples were preserved in 10% formalin, transported to a laboratory in Quito, and stored at 4°C until examination by light microscopy. Eggs were concentrated by using the formalin-ether technique. In addition, 120 fecal samples from Afro-Ecuadorian and mestizo persons were examined. The villagers were informed of the study in their local Chapalache language by community health community workers. The ethical committee of the Central University approved this study.

Duodendoscopy was performed in 4 patients by a gastroenterology specialist to examine the biliary liquid; the microscopy of this liquid showed eggs identical to those found in their feces. These patients received praziquantel (75 mg/kg in 3 doses/3 d), and were purged with 10 mg of bisacodilo. Fecal samples were collected and examined for worms as previously described (*12*). Recovered worms were fixed with 10% formalin, stained with Diff-Quik fixative (Sysmex, Kobe, Japan), and identified by comparing their morphologic features to known adult *Clonorchis* and *Opisthorchis* spp. worms. Community health workers collected and examined the livers of 3 cats and 3 dogs from 1 of the 3 communities. All 6 livers had eggs and high numbers of adult parasites in the bile ducts. Adult parasites were stained, and microscopic studies showed them to be identical to those in the human specimens.

**Figure 1:**
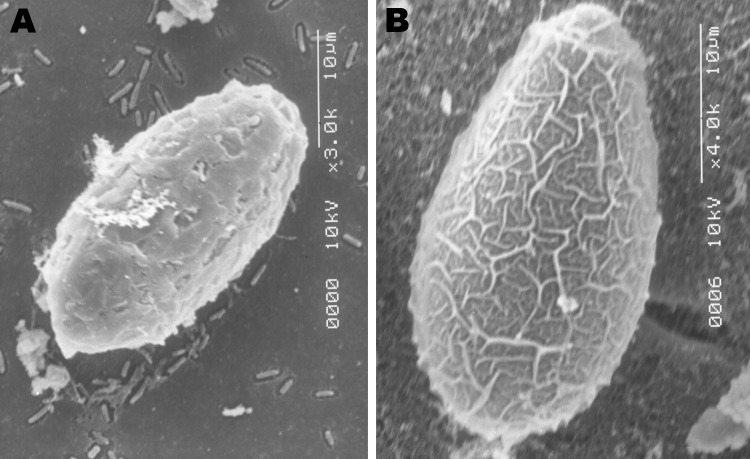
Scanning electron microscopy images of A) an egg of the Ecuadorian *Amphimerus* spp. trematode (original magnification ×3) obtained from a human and B) an egg of the Asian *Clonorchis sinensis* trematode (original magnification ×4). Although the size is similar, the pattern of the surface is different, thus differentiating the 2 genera.

A total of 71 (24%) of the 297 fecal samples from the indigenous Chachi were positive for *Opisthorchiidae* eggs (Table). In contrast all 120 samples from Afro-Ecuadorian and mestizo persons were negative. Eggs were yellow-brown and measured 28–33 μm ×12–15 μm (n = 20). The operculum and the shoulders, however, were not prominent as they are in *Clonorchis* and *Opisthorchis* eggs. Occasionally, a small knob, but most frequently a curved spine, was seen on the abopercular end. Although, by light microscopy, the shape and size of the eggs resembled that of the other liver flukes*,* the patterns of the eggshell surface were distinct when viewed with SEM (Figure 1). This observation is corroborated with published photographs (*3*).

**Table:** Prevalence of *Amphimerus* eggs in feces from 3 villages, Ecuador

Village	Total population	No. samples examined	No. (%) positive	Distance to the coast, km
1	116	82	28 (34.1)	120
2	248	86	23 (26.7)	91
3	253	129	20 (15.5)	85
Total	617	297	71 (23.9)	

**Figure 2:**
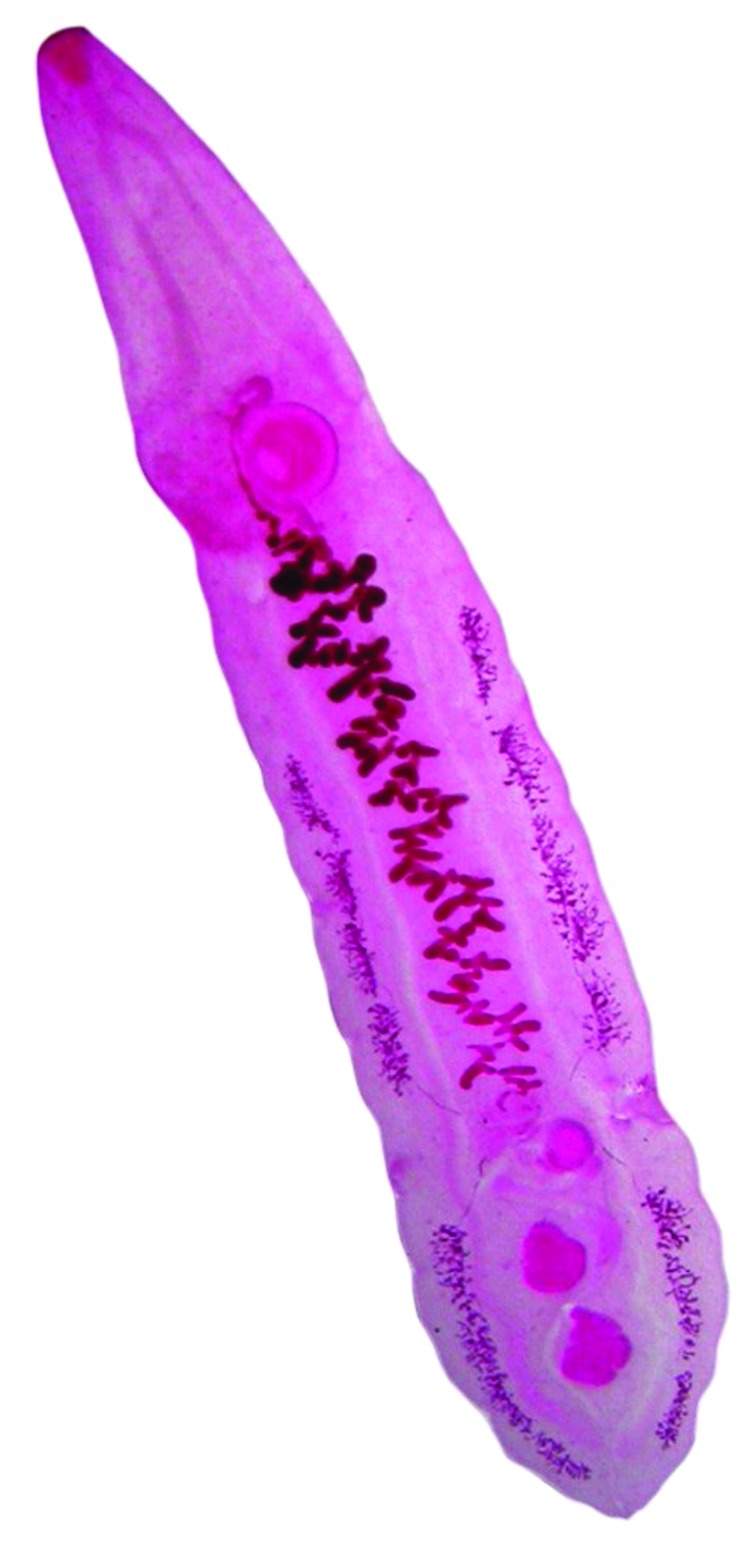
. *Amphimerus* spp. adult parasite (10.1 mm) recovered from a human, Ecuador.

After participants were treated with praziquantel, a total of 8 worms were recovered from 4 human participants and dozens from cat and dog livers; all were placed in saline. The worms were delicate, leaf-shaped, elongated, and red-pink and measured 8–13.6 mm long (average 10.2 mm) × 0.5–1.1 mm wide (n = 15). After a few minutes, the worms coiled in an S shape and became transparent or whitish. Once stained, the following features were observed: 1) the vitellaria divided into an anterior and posterior group with the posterior group extending the level of the posterior testis; 2) a ventral sucker larger than oral sucker; and 3) 2 rounded testes (Figure 2). On the basis of these morphologic characteristics of the adults and the SEM findings of the eggs, the parasite was identified as *Amphimerus* spp.

## Conclusions

Our study demonstrates that the liver fluke of the genus *Amphimerus* can infect humans. We found a high prevalence (15.5%–34.1%) of infection with *Amphimerus* spp. trematodes in the surveyed communities (Table). Samples from the Afro-Ecuadorian and mestizo population were all negative for *Opisthorchiidae* eggs. *Amphimerus* spp. trematodes are believed to be transmitted, as are the other members of the *Opisthorchiidae* family, by ingestion of raw or undercooked fish (*2*). In our survey, most Chachis reported eating smoked fish caught in the rivers. Food sharing is more common among Chachi than Afro-Ecuadorians and mestizo families (*13*). Notably, the most remote village (120 km inland from the coast) had the highest prevalence. Our results suggest that *Amphimerus* spp. flukes are zoonotic pathogens transmitted by domestic animals living with humans.

Amphimeriasis should be considered an endemic liver fluke infection among residents of this Chachi population in Ecuador. Further studies are needed to determine the complete epidemiology and geographic distribution of infection in this region, as well as in other provinces of Ecuador where freshwater fish is eaten undercooked or where the same tropical ecology is found. For example, the Amazonian region has indigenous groups where other foodborne trematodiasis-like paragonimiasis are endemic (*14*). *Amphimerus* spp. flukes infecting domestic and wild animals have been reported from Ecuador’s neighboring countries as well as from Central and North America. The existence of undiscovered foci of human infections is possible.

In 1971, Yamaguti (*1*) suggested that a parasite previously reported in Ecuador (*8*) as *O. guayaquilensis* might in fact be *Amphimerus* spp. Subsequently, publications referred to this parasite as *A. guayaquilensis* (*5**,**7*); however, the accuracy of this reclassification is unclear. Molecular analysis could help clarify the ambiguities in genus/species identification of *O. guayaquilensis* and the conspecific species of *Amphimerus* (*15*).

We have much to learn about the pathology and epidemiology of *Amphimerus* spp. flukes. For example, nothing is known about the clinical and pathologic significance of infections with this parasite. Praziquantel eliminated the parasites in these patients, but whether the dose and treatment time were adequate are unknown. Additionally, little is known about epidemiologic factors responsible for the differences in the number of infections among the different population groups. Future studies can help determine the direct and indirect public health implications of this new foodborne zoonosis.
